# Supplementary material for: Metabolome Characteristics of Liver Autophagy Deficiency under Starvation Conditions in Infancy
Source: Nutrients. 2021 Aug 29;13(9):3026. doi: 10.3390/nu13093026 (PMC8470362; doi:10.3390/nu13093026)
Supplement: Supplementary file 1 [file nutrients-13-03026-s001.zip › nutrients-1332119-supplementary.pdf]

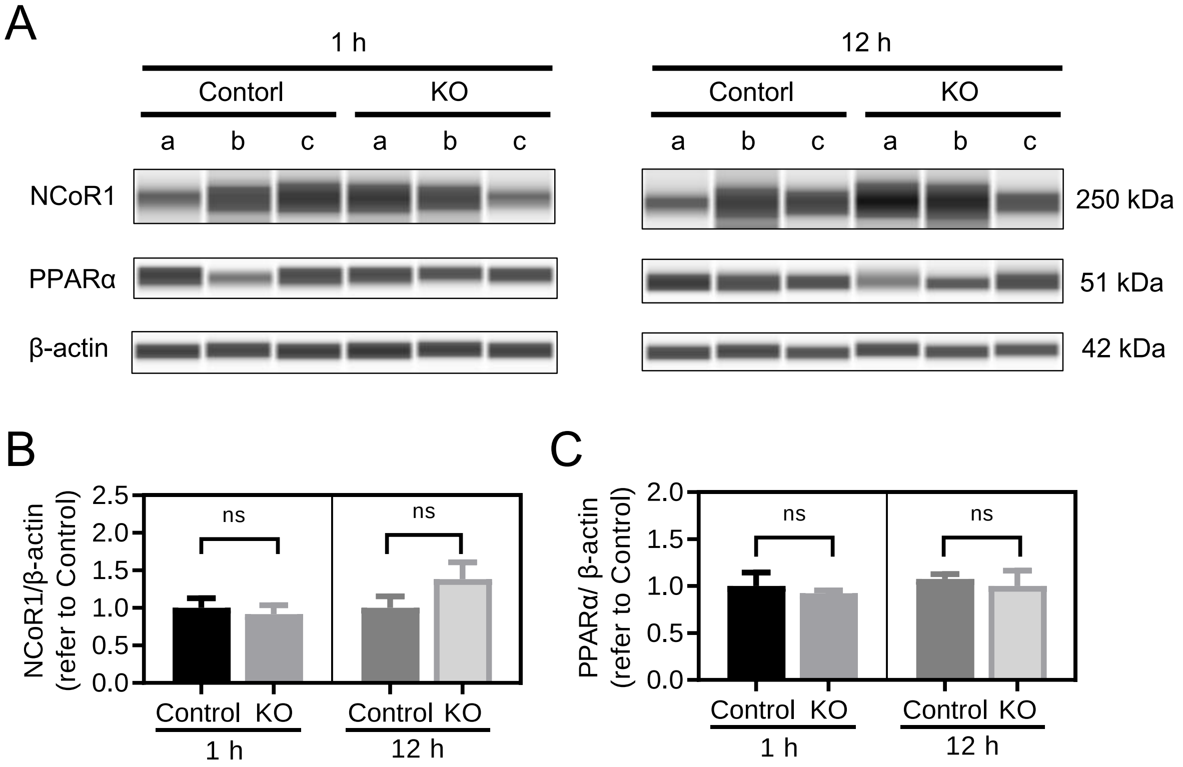

Supplementary Figure S1. The automated Western blotting analysis of NCoR1 and PPARα in the liver. (A) The protein expression of NCoR1 and PPARα in liver extract of Control and KO mice after 1 and 12 h of starvation (n = 3 at each starvation time point). The expression of NCoR1 (B) and PPARα (C) refer to Control mice at 1 and 12 h of starvation were shown in graphs. β-actin was used as an internal control. Each bar is expressed as the mean ± SEM. P, Student's t-test; KO: liver-specific *Atg5*-deficient; NCoR1: nuclear receptor co-repressor 1; PPARα: peroxisome proliferator-activated receptor α.

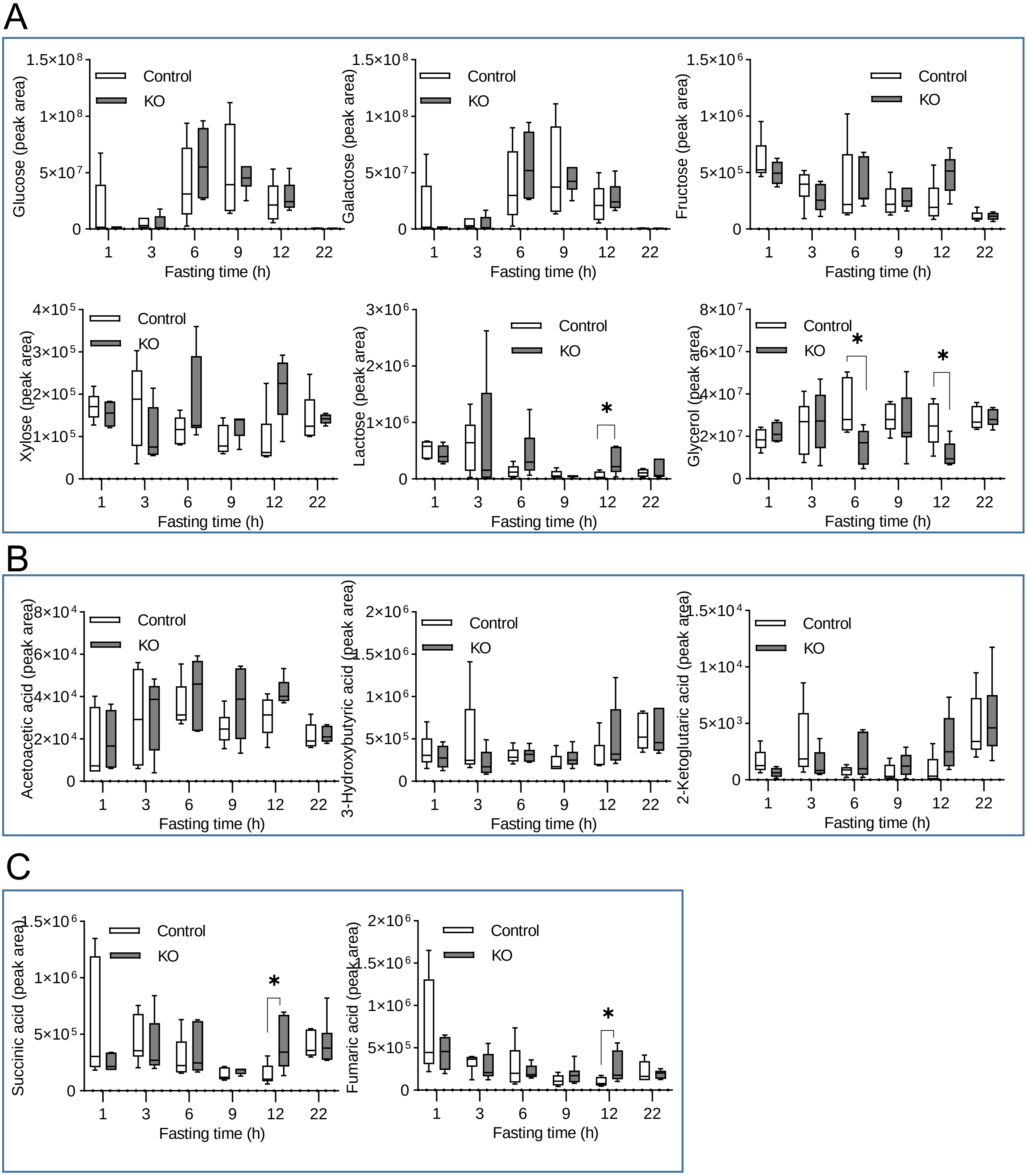

Supplementary Figure S2. **The trajectory course of serum (A) carbohydrates and glycerol , (B) ketone bodies and (C) Succinic acid and Fumaric acid by the GC-MS/MS analysis with 22-h starvation survivors**

The numbers of mice (starvation time) were as follows: 5 (1 h), 6 (3 h), 5 (6 h), 6 (9 h), 6 (12 h), and 5 (22 h) Control mice and 4 (1 h), 5 (3 h), 7 (6 h), 7 (9 h), 5 (12 h), and 6 (22 h) KO mice. The concentration of metabolites was measured as the peak area of the calibration curve, and the concentration at each starvation time was compared between the groups. Boxes represent the interquartile range (25th to 75th percentiles), and lines within the boxes are the median; error bars represent the 25th percentile minus 1.5 time interquartile range (IQR) and the 75th percentile plus 1.5 times IQR. Mann-Whitney U test; **P* <0.05, ***P* <0.01. KO: liver-specific *Atg5*-deficient.

A

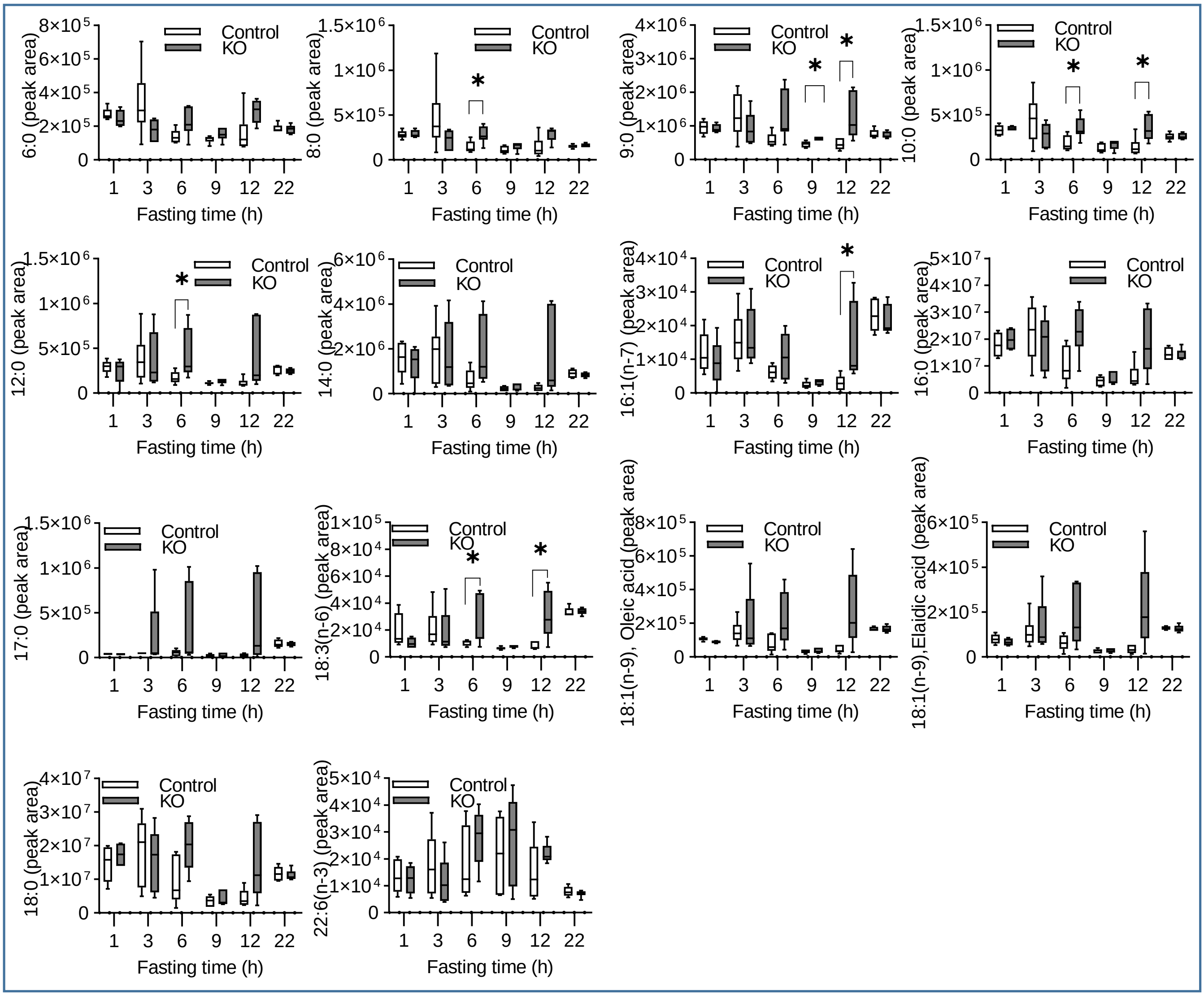

Supplementary Figure S3. **The trajectory course of serum FFAs with the GC-MS/MS analysis with 22-h starvation survivors**

The numbers of mice (starvation time) were as follows: 5 (1 h), 6 (3 h), 5 (6 h), 6 (9 h), 6 (12 h), and 5 (22 h) Control mice and 4 (1 h), 5 (3 h), 7 (6 h), 7 (9 h), 5 (12 h), and 6 (22 h) KO mice. The concentration of metabolites was measured as the peak area of the calibration curve, and the concentration at each starvation time was compared between the groups. Boxes represent the interquartile range (25th to 75th percentiles), and lines within the boxes are the median; error bars represent the 25th percentile minus 1.5 time interquartile range (IQR) and the 75th percentile plus 1.5 times IQR. Mann-Whitney U test; *P <0.05, **P <0.01. KO: liver-specific *Atg5*-deficient.

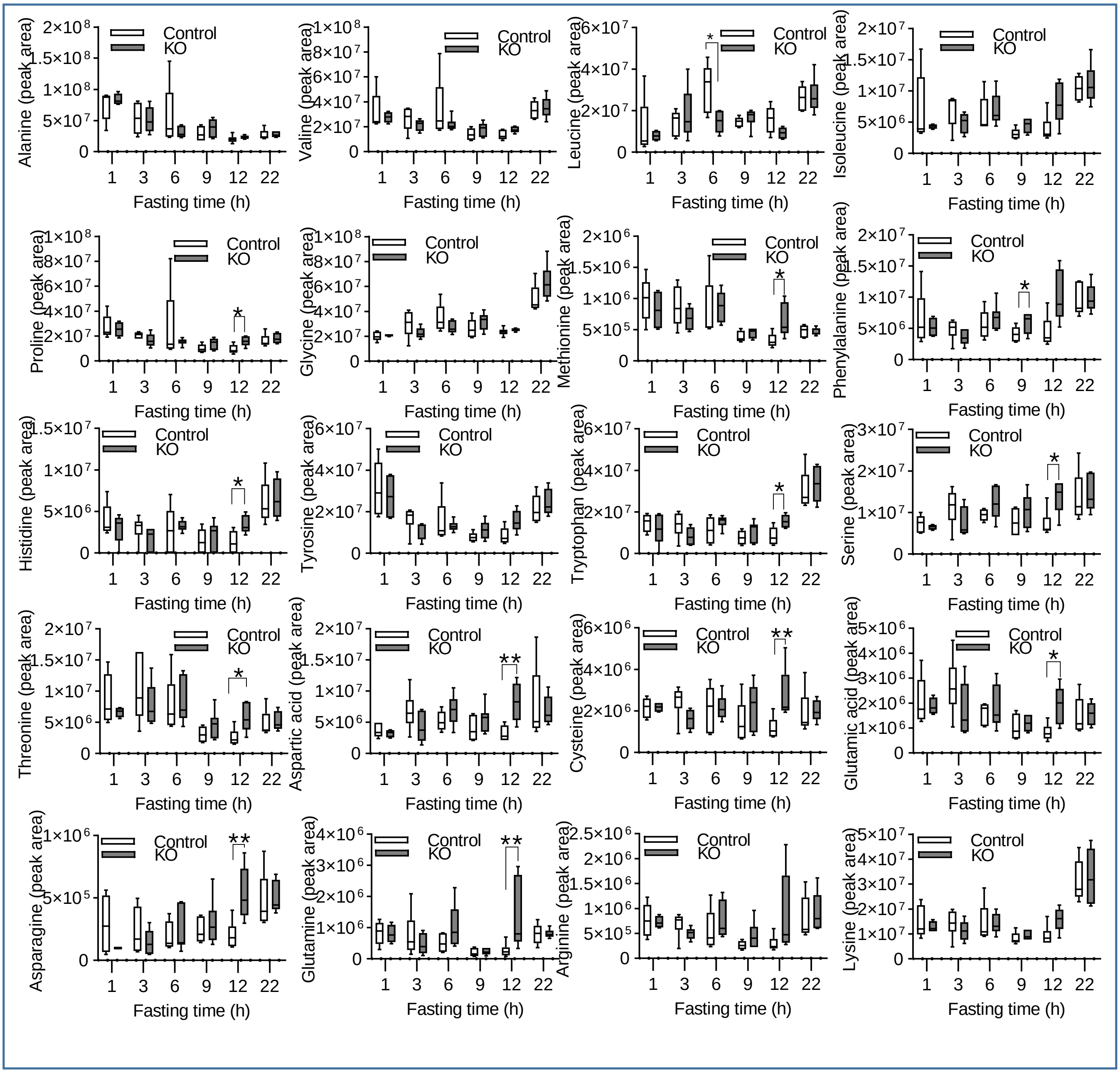

Supplementary Figure S4. The trajectory course of serum 20 protein-cording amino acids with GC-MS/MS analysis with 22-h starvation survivors

The numbers of mice (starvation time) were as follows: 5 (1 h), 6 (3 h), 5 (6 h), 6 (9 h), 6 (12 h), and 5 (22 h) Control mice and 4 (1 h), 5 (3 h), 7 (6 h), 7 (9 h), 5 (12 h), and 6 (22 h) KO mice. The concentration of metabolites was measured as the peak area of the calibration curve, and the concentration at each starvation time was compared between the groups. Boxes represent the interquartile range (25th to 75th percentiles), and lines within the boxes are the median; error bars represent the 25th percentile minus 1.5 time interquartile range (IQR) and the 75th percentile plus 1.5 times IQR. Mann-Whitney U test ; *P <0.05. KO: liver-specific *Atg5*-deficient.

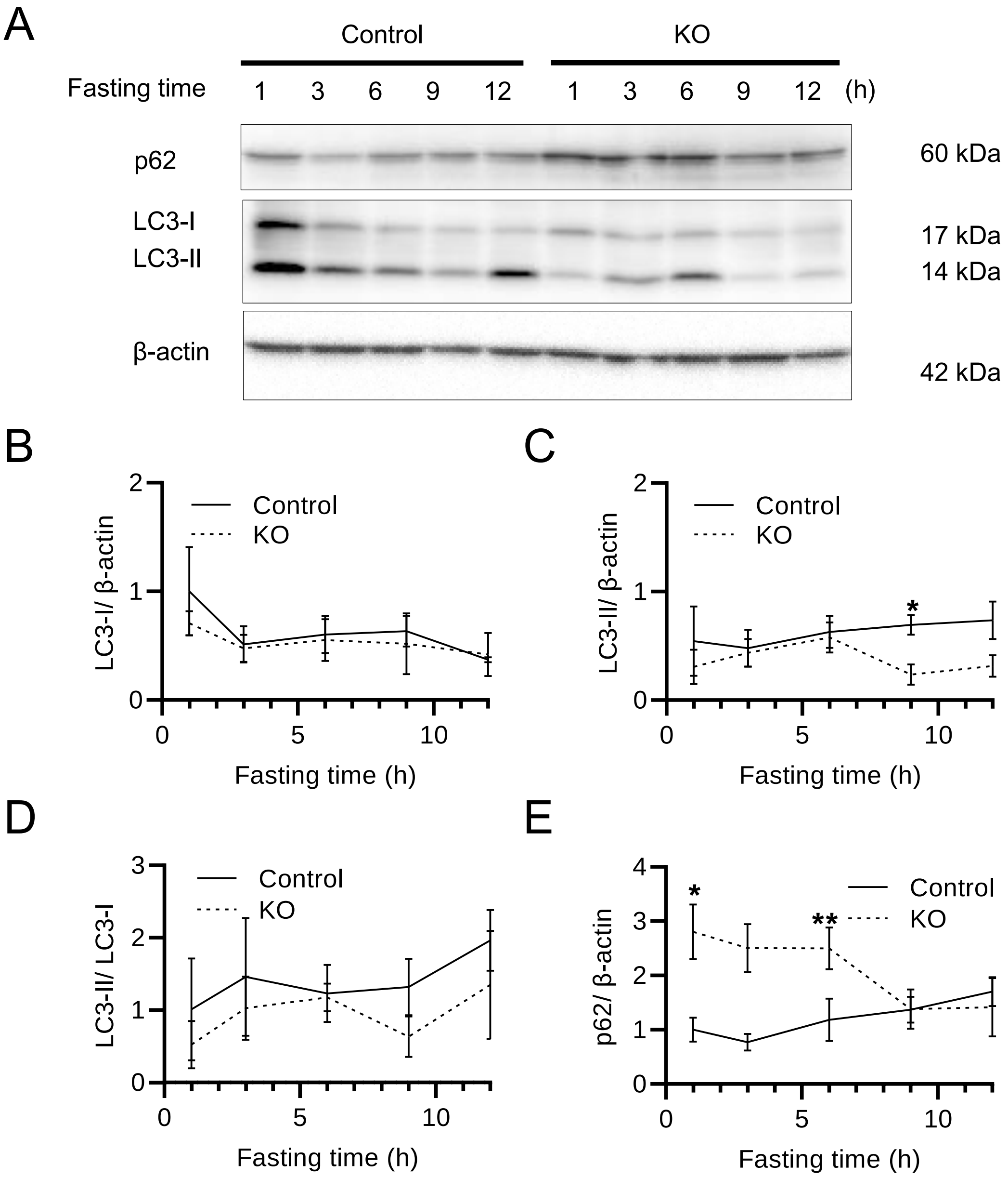

Supplementary Figure S5. The Western blotting analysis of LC3, and p62 in extract protein of muscles.

(A) The protein expression of LC3-I, LC3-II, and p62 in muscle after 1–12 h starvation (n = 4 at each starvation time). β-actin was used as an internal control. The full-length blots are presented in Supplementary Figure S7. The relative expression of LC3-I (B) and LC3-II (C), LC3-II/LC3-I ratio (D), and p62 (E) are shown in graphs. Each bar is expressed as the mean ± SEM. Student's t-test;**P* <0.05, ***P* <0.01. KO: liver-specific *Atg5*-deficient; LC3: microtubule-associated protein 1A/1B-light chain 3; LC3-I: cytosolic form of LC3; LC3-II: phosphatidylethanolamine conjugate form of LC3.

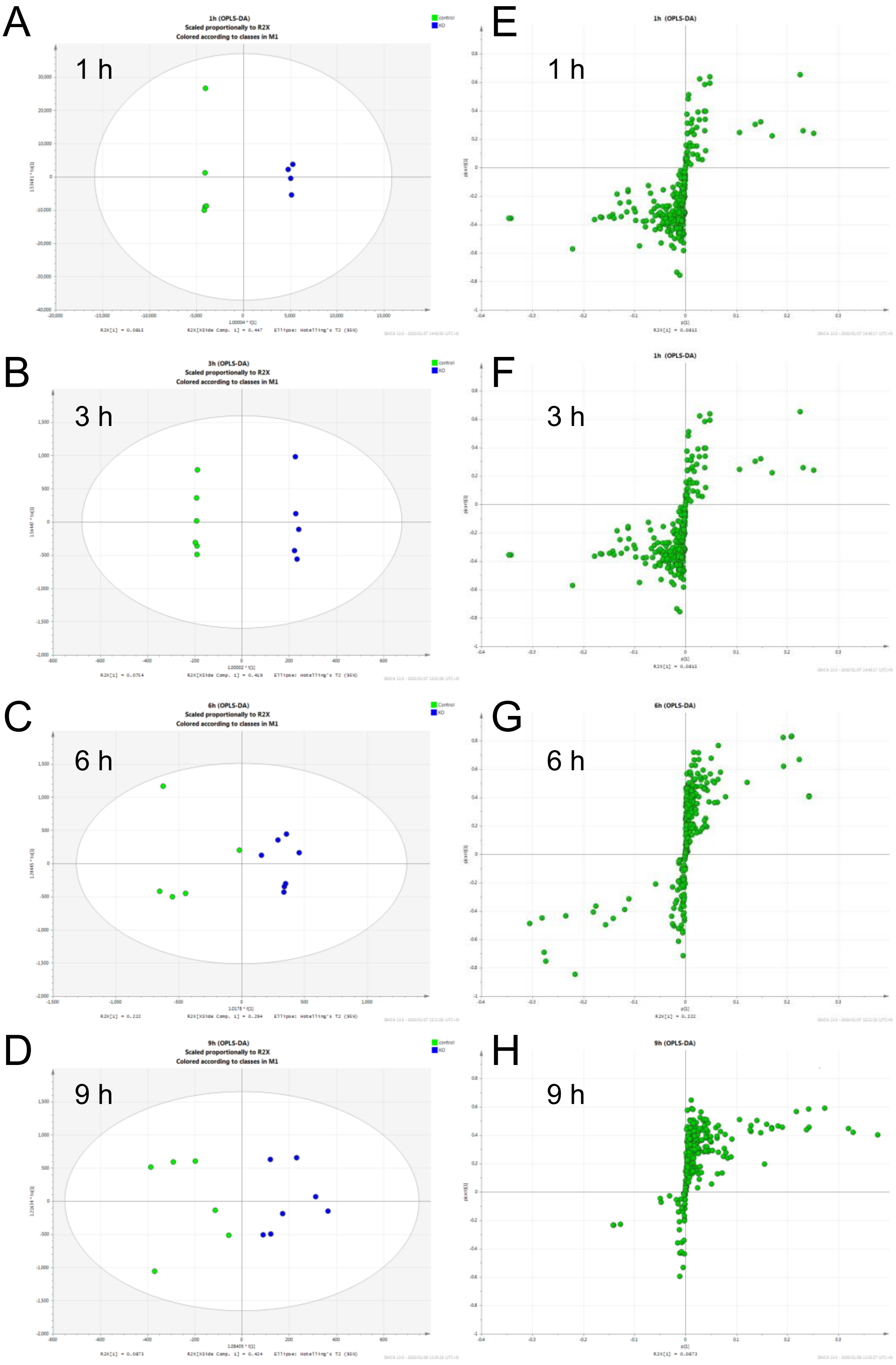

Supplementary Figure S6. **Multivariate statistical and pathway analyses (OPLS-DA) of serum metabolites at each starvation timepoint**

(A-D) Score scatter plots of serum metabolites from the liver-specific Atg5-deficient (KO, blue dot) and Control groups (green dot) at 1 h (A), 3 h (B), 6 h (C), and 9 h (D) of starvation. A differential metabolic pattern was noted between KO and Control mice by the two plots clearly separated on the score scatter plot. (E-H) S-plots of serum metabolites from the KO and Control groups at 1 h (E), 3 h (F), 6 (G), and 9 h (H) of starvation. The number of significant metabolites with |p (corr)| > 0.7 at each timepoint was less than that of 12-hour starvation. OPLS-DA: orthogonal partial least squares discriminant analysis; KO: liver-specific *Atg5*-deficient.

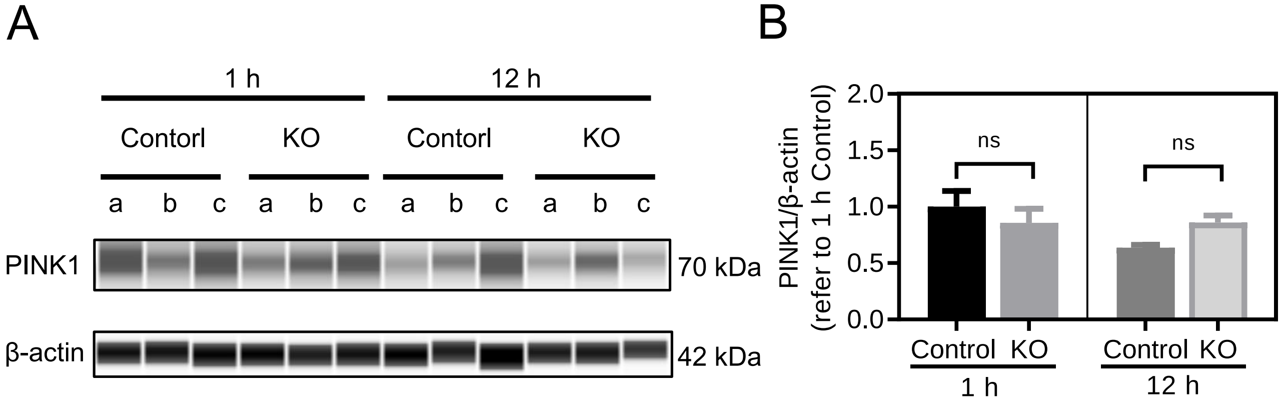

Supplementary Figure S7. The automated Western blotting analysis of PINK1 in the liver. (A) The protein expression of PIKN1 in liver extract of Control and KO mice after 1 and 12 h of starvation (n = 3 at each starvation time point). (B) The expression of PINK1 and at 1 and 12 h of starvation refer to 1 h Control mice were shown in graphs. β-actin was used as an internal control. Each bar is expressed as the mean ± SEM. P, Student's t-test; KO: liver-specific *Atg5*-deficient; PINK1: PTEN-induced putative kinase 1.

Supplementary Table S1. Metabolites significantly differed according to S-plot analysis Control vs. KO mice at each starvation time.

starvation duration	A	N	R^2X (cum)	R^2Y (cum)	Q^2 (cum)
1 h	1+5+0	9	0.986	0.999	0.116
3 h	1+7+0	11	0.989	0.999	0.161
6 h	1+2+0	12	0.694	0.850	0.433
9 h	1+2+0	13	0.769	0.796	0.0238
12 h	1+4+0	11	0.960	0.990	0.163

OPLS-DA: orthogonal partial least squares discriminant analysis; KO: liver-specific *Atg5*-deficient.

Supplementary Table S2. Metabolites significantly differed according to S-plot analysis Control vs. KO mice at each starvation time.

Var ID (Primary)	metabolite	p[1]	p(corr)
1 h, control > KO			
107	Dihydrouracil-TMS	-0.01141	-0.75722
388	Oleic acid-TMS	-0.01732	-0.73572
3 h, control > KO			
155	O-Phosphoethanolamine-3TMS	-0.01174	-0.77125
253	2-Deoxy-glucose-4TMS	-0.02405	-0.70130
6 h, control < KO			
312	Allose-meto-5TMS	0.20847	0.83128
322	Glucose-meto-5TMS	0.20847	0.83126
319	Galactose-meto-5TMS	0.20769	0.82792
313	Mannose-meto-5TMS	0.19118	0.82189
309	N-Acetylglutamine-3TMS	0.06403	0.76646
355	Gluconic acid-6TMS	0.01658	0.71804
60	Octanoic acid-TMS	0.02562	0.71698
6 h, cotrol > KO			
62	2-Aminoethanol-3TMS	-0.21632	-0.84693
63	Leucine-2TMS	-0.27356	-0.75216
40	Glyceraldehyde-meto-2TMS	-0.00423	-0.71479
12 h, control < KO			
243	Ribonic acid-5TMS	0.02938	0.79801
199	Homocysteine-3TMS	0.01356	0.78809
265	Dopamine-3TMS	0.04672	0.78808
137	4-Aminobutyric acid-3TMS	0.03628	0.78633
150	Creatinine-3TMS	0.09444	0.78323
128	3-Aminoglutaric acid-2TMS	0.15668	0.78298
130	Aspartic acid-3TMS	0.14681	0.78063
451	Guanosine-5TMS	0.01388	0.77790
169	3-Aminoglutaric acid-3TMS	0.01918	0.77303
226	Orotic acid-3TMS	0.01075	0.77204
200	Asparagine-3TMS	0.03729	0.75772
241	Homovanillic acid-2TMS	0.00715	0.75634
277	Tagatose-meto-5TMS	0.00947	0.75407
80	Catechol-2TMS	0.00375	0.75161
206	Taurine-3TMS	0.17443	0.75102
276	1,5-Anhydro-glucitol-4TMS	0.01269	0.75064
12 h, control < KO			
263	Ornithine-4TMS	0.17683	0.74577
403	Cystine-4TMS	0.02478	0.74305
122	Threitol-4TMS	0.01018	0.74165
396	Tryptophan-3TMS	0.17128	0.73822
196	Lyxose-meto-4TMS	0.02697	0.73355
175	Glutamic acid-3TMS	0.06575	0.73231
431	Serotonin	0.03702	0.73056
247	Dihydroorotic acid-3TMS	0.01064	0.72521
190	N-Acetylaspartic acid-2TMS	0.01014	0.72162
86	Serine-3TMS	0.16310	0.72124
440	Batyl alcohol-2TMS	0.00919	0.71959
333	Glucuronic acid-meto-5TMS	0.05643	0.71790
437	Inosine-4TMS	0.06958	0.71788
328	Mannitol-6TMS	0.02347	0.71638
339	Galacturonic acid-meto-5TMS	0.05714	0.71597
358	Glucaric acid-6TMS	0.01198	0.71474
359	Allantoin-3TMS	0.01721	0.71434
223	Aconitic acid-3TMS	0.00594	0.71427
144	Threonic acid-4TMS	0.07346	0.71411
189	2-Ketoglutaric acid-oxime-3TMS	0.00241	0.71336
348	Galacturonic acid-meto-5TMS	0.05448	0.71330
208	Taurine-13C2-3TMS	0.13807	0.71319
113	Citramalic acid-3TMS	0.00809	0.71142
149	2-Hydroxyglutaric acid-3TMS	0.02619	0.70978
242	1-Methylhistamine dihydrochloride	0.02127	0.70818
106	Decanoic acid-TMS	0.02908	0.70778
300	Allantoin-4TMS	0.02817	0.70701
254	2-Aminopimelic acid-3TMS	0.05809	0.70661
74	Proline-2TMS	0.16142	0.70607
344	Glucuronic acid-meto-5TMS	0.02416	0.70395
454	Maltose-meto-8TMS	0.05947	0.70199
447	Lactose-meto-8TMS	0.00384	0.70140
452	Trehalose-8TMS	0.11327	0.70117
91	Threonine-3TMS	0.11286	0.70069
457	Maltose-meto-8TMS	0.05003	0.70019
12 h, control > KO			
400	Tryptamine-3TMS	-0.04133	-0.82715

KO: liver-specific *Atg5*-deficient; TMS: trimethylsilyl.
